# Toward Real‐Time Backscatter Coefficient Estimation Incorporating the U‐Net Segmentation and an In Vivo Reference Target

**DOI:** 10.1002/jum.16750

**Published:** 2025-06-25

**Authors:** Yuning Zhao, Zhengchang Kou, Conn Louie, Rita J. Miller, Gregory J. Czarnota, Michael L. Oelze

**Affiliations:** ^1^ Beckman Institute for Advanced Science and Technology University of Illinois at Urbana‐Champaign Urbana Illinois United States; ^2^ Department of Electrical and Computer Engineering University of Illinois at Urbana‐Champaign Urbana Illinois United States; ^3^ Department of Medical Biophysics and Radiation Oncology University of Toronto Toronto Ontario Canada; ^4^ Department of Imaging Research and Radiation Oncology Sunnybrook Health Sciences Center Toronto Ontario Canada; ^5^ Carle Illinois College of Medicine University of Illinois at Urbana‐Champaign Urbana Illinois United States

**Keywords:** backscatter coefficient (BSC), breast cancer, U‐Net

## Abstract

Quantitative ultrasound using spectral‐based techniques, like the backscatter coefficient (BSC), have demonstrated capabilities for tumor characterization and therapy monitoring. The incorporation of an in situ calibration target, that is, a small titanium bead, can provide more consistent BSC estimates. For analyzing tumors, BSC estimation traditionally relies on manual tumor segmentation and calibration bead detection, a time‐consuming and skill‐dependent task. This study utilizes a U‐Net model for automatic BSC estimation by integrating identification of a titanium calibration target embedded in rabbit mammary tumors with automatic segmentation, enabling real‐time applications. The U‐Net model demonstrated strong segmentation performance, achieving a Dice score of 0.86. Performance metrics demonstrated reliable BSC parameter estimation, with relative errors of 17.87% for effective scatter diameter (ESD) and 9.95% for effective attenuation concentration (EAC) when comparing automated segmentation to manual segmented tumors, highlighting its potential for accurate, real‐time tumor diagnostics and therapy monitoring in clinical practice.

**Abbreviations**
BSC: backscatter coefficient
BSF: bead scattering function
DSC: Dice similarity coefficient
EAC: effective attenuation concentration
ESD: effective scatter diameter
IQ: in‐phase and quadrature
LABC: locally advanced breast cancer
NAC: neoadjuvant chemotherapy
NCC: normalized cross‐correlating
QUS: quantitative ultrasound
RCA‐IUnet: residual cross‐spatial attention inception U‐Net
RF: radiofrequency
ROI: region of interest
SLN: sentinel lymph node

In this particular application, we are utilizing quantitative ultrasound (QUS) with spectral‐based techniques for characterizing breast cancer. Breast cancer represents the most common form of cancer among women. Data from the Global Cancer Observatory in 2022 indicates that breast cancer accounts for 23.8% of all cancer incidences among females and 15.4% of all cancer‐related mortality.^1^ Furthermore, breast cancer ranks as the leading cause of new cancer cases among U.S. females, with an incidence rate of 119.2 per 100,000 women.^2^ Spectral‐based QUS is a tissue characterization technique that examines the frequency content of the radiofrequency (RF) or in‐phase and quadrature (IQ)^3^ backscattered ultrasound signals from tissues, often by estimating the backscatter coefficient (BSC) versus frequency of the tumor and associated spectral parameters. The QUS method has been widely applied to breast cancer applications. For example, Nam et al^4^ demonstrated that the BSC and derived parameters could differentiate carcinomas from fiboradenomas. Tadayyon et al^5^ demonstrated the ability of QUS parameters to provide an early prediction of tumor response to neoadjuvant chemotherapy (NAC) in patients with locally advanced breast cancer (LABC). Research from Osapoetra et al^6^ found that QUS spectral parametric imaging along with texture and texture‐derivate analyses have high potential for rapid, accurate, and non‐invasive characterization of breast lesions. Taleghamar et al^7^ assessed the use of QUS imaging and machine learning to forecast breast cancer response to chemotherapy even before treatment. Analysis of ultrasound data from 181 patients led to a biomarker that predicts response with 85.4% accuracy, surpassing traditional clinical predictions. Argueta‐Lozano et al^8^ assessed the inter‐ and intra‐operator variability of the BSC for breast mass characterization, using a regularized PEQUS strategy applied clinically for the first time. Klimonda et al^9^ used QUS to detect tumor response to NAC at an early stage of treatment. These findings highlight the potential of QUS imaging in breast cancer diagnostics and pre‐treatment cancer characterization.

Most BSC estimation methods, including the planar reflector method,^10^, ^11^ and the reference phantom technique^12^ rely on external references, which are limited in accounting for the attenuation and transmission losses from the transducer to the region of interest (ROI).^13^ To correct the transmission and attenuation losses for BSC estimation, Nguyen et al^14^ introduced a small metallic bead, that is, titanium (Ti), as an *in situ* reference. The bead could act as a radiological marker and as an internal reference or calibration target for QUS techniques. Clinically, biopsy clips or markers are routinely implanted into breast tissue to accurately identify and track areas undergoing treatment. In patients with node‐positive breast cancer undergoing NAC, placing a marker within a positive lymph node facilitates accurate surgical removal of the targeted node during sentinel lymph node (SLN) surgery, thus improving surgical staging accuracy.^15^, ^16^ The biopsy clip method would enable non‐invasive, continuous monitoring of tissue properties throughout treatment, supporting more precise, real‐time assessment of therapeutic responses.

Because the reference material is in the sample medium, ultrasound signals reflected from the bead share similar transmission and attenuation losses with adjacent regions in the sample. Therefore, the in situ calibration approach using a small metallic bead can provide a calibration spectrum that also approximately accounts for transmission and attenuation losses near the bead location.

Further investigations into the in situ bead reference method by Cario et al^17^ demonstrated that consistent calibration spectra from the bead could be obtained through averaging spectra backscattered from across the bead surface even if the bead was larger than the beamwidth. A recent study by Zhao et al^18^ quantified the ability of the in situ reference method to correct for the attenuation loss when evaluating the BSC parameters such as effective scatter diameter (ESD) and effective attenuation concentration (EAC) in rabbit breast tumors. In that study, the performance of the bead reference was found to be superior to the reference phantom method when estimating the BSC from tumors with and without a lossy layer placed on top of the tumors.

The integration of tumor BSC calculations into clinical practice is instrumental for enhancing diagnostic accuracy and treatment monitoring. However, when using the *in situ* bead calibration target, identification of a frame containing the bead and the actual bead location in the frame is required. For external reference techniques, there is no need to identify a target in situ. Furthermore, current methods often require manual or semi‐automated segmentation of an ROI, which is time‐consuming and prone to variability. Segmentation is necessary to isolate tumor regions for BSC analysis and to automatically locate the bead reference in a frame, yet this step can hinder the real‐time application of QUS techniques.

Advanced deep learning methods, such as U‐Net, have been developed to automate tumor segmentation in ultrasound images, facilitating the potential for real‐time analysis.^19^ The use of U‐Net for the breast tumor ultrasound image segmentation has been extensively studied, yielding significant advancements. For example, Zhang et al^20^ achieved a dice similarity coefficient of 0.898 for segmentation of mass regions. Punn and Agarwal^21^ developed a deep learning based model, residual cross‐spatial attention inception U‐Net (RCA‐IUnet), specifically for breast tumor segmentation in ultrasound imaging. Yang et al^22^ proposed a multi‐task framework that integrated tumor segmentation based on U‐Net with classification tasks for breast ultrasound images, achieving an average accuracy of 97% for benign and malignant diagnoses. The efficient use of data with a U‐Net allows it to be trained end‐to‐end from very few images and outperforms prior methods, particularly in situations where the available training data is scarce. With the incorporation of the segmentation workflow, we could facilitate consistent and immediate BSC calculations from tumor regions. Additionally, these methods assist in identifying a calibration bead within an image, which enables rapid QUS analysis during diagnostic and therapeutic procedures.

This study presents a novel approach focusing on automatic BSC evaluation through tumor segmentation and automatic identification of the in situ bead reference, rather than on segmentation itself. By leveraging advanced segmentation as a tool rather than an end, our method aims to streamline BSC parameter estimation, reducing the dependency on manual input and accelerating the integration of QUS into clinical workflows. This approach not only improves the accuracy and consistency of tumor characterization and facilitates localization of the in situ reference target but also paves the way for real‐time QUS imaging modes, enhancing the overall impact of ultrasound in breast cancer management.

## Materials and Methods

### In Vivo Tumor Data Collection

The protocol for the rabbit experiments was approved by the University of Illinois at Urbana‐Champaign Institutional Animal Care and Use Committee (IACUC protocol 20087). A total of 21 rabbits, each with 2 tumors, 1 on the right side and 1 on the left side, were scanned in this study. A 2‐mm diameter spherical titanium bead was implanted into each tumor to serve as an *in vivo* reference target. A single rabbit experiment spanned from 3 to 5 weeks and comprised 3 stages:

1. Tumor injection and growth: VX2 tumor fragments were injected into the mammary fat pad of a 2 kg female New Zealand White Rabbit, under anesthesia using 2% isoflurane. VX2 is an anaplastic squamous cell carcinoma that has been used as a tumor model in various tissues, such as breast, liver, lung, and neck. It is characterized by a fast growth rate, hypervascularity, and is easily propagated in skeletal muscle for injection elsewhere. The rabbit breast VX2 model is a good model of breast cancers due to the rapid growth of VX2 tumors and similarity to human breast cancer and has been used in numerous imaging and therapy studies for many years.^23^, ^24^, ^25^, ^26^ This is often used instead of rats or mice because the size of the animal allows conventional imaging devices to be tested. Therefore, we chose the VX2 model because of the animal size; it has been established in the literature as a model for human breast cancer, and we have familiarity with the model. The tumor growth was monitored for the following 2–3 weeks until the tumor diameter reached approximately 2.5 cm.
2. Bead injection: When the tumor reached approximately 2.5 cm in diameter, a 2‐mm spherical Ti bead was implanted into the tumor area with a 12‐gauge stainless steel needle after shaving and disinfecting the area.
3. Imaging and data collection: Both on the injection day and 3 days after the bead injection, the tumor with the embedded bead was imaged using a SonixOne ultrasound imaging scanner equipped with an L14‐5/38 transducer (center frequency: 7.2 MHz, −6 dB bandwidth of 70%). The SonixOne machine was operated at 6.6 MHz, and the RF data were sampled at 40 MHz, corresponding to an axial spatial resolution of approximately 0.385 mm. Additionally, 8 of the rabbits were in a separate longitudinal study and were scanned weekly on days 7, 17, 24, and so on, continuing until the tumor became too large, at which point the rabbits were euthanized. Images were captured with and without a sample of pork belly, around 1.5–2 cm thick, purchased from a local supermarket and placed on top of the tumor to simulate a fat layer. The transducer was scanned across the tumor by free hand translation. This resulted in a volumetric scan of the tumor through stacking of individual frames of the tumor. RF data and corresponding video files were recorded via the SonixOne ultrasound machine for postprocessing and BSC estimation. A scan of an external reference phantom was obtained for each rabbit scan. Because we do not have full access to the programming interface and hardware of the SonixOne scanner, we had to conduct all analysis and segmentation in postprocessing and not in real time.

### U‐Net Architecture

**Figure 1:**
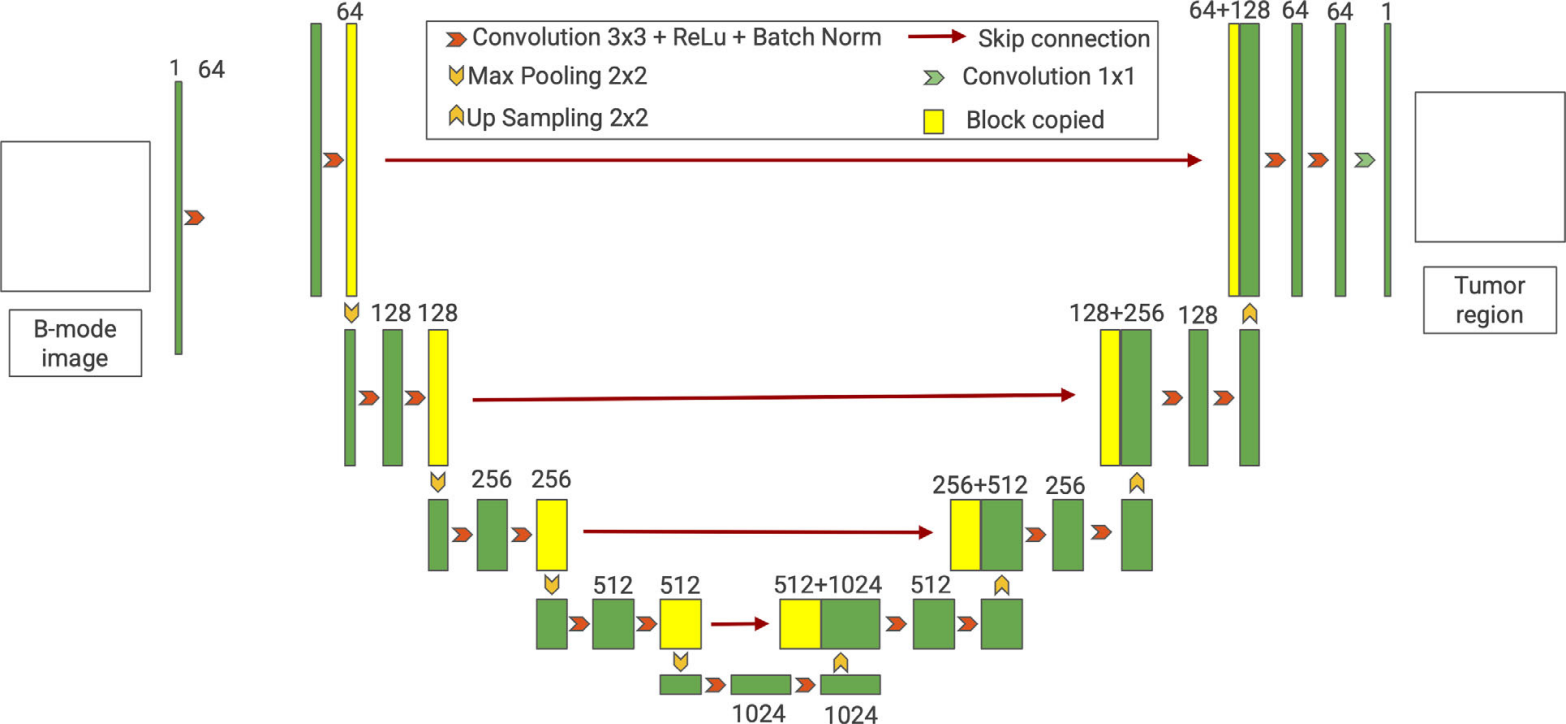
Architecture of the U‐Net network for tumor segmentation.

As shown in Figure 1, this network was comprised of a sequence of 9 layers, wherein the dimensional configuration progressively escalated from 64 to 128, 256, 512, and peaked at 1024, before symmetrically descending back to 64 in the decoding phase and finally a single channel for output. Each layer within this architecture underwent a convolution operation employing a 3 × 3 kernel. Subsequent to this convolution, the network applied the Rectified Linear Unit (ReLU) activation function and Batch Normalization in a consistent manner across all layers.

The network was designed to process a B‐mode image, represented with a single channel, as its input. The output generated by the U‐Net was a binary mask delineating the tumor region, encoded over 2 channels. Within this output, the presence of a tumor was denoted by a value of 1, while the absence was indicated by 0.

For training the U‐Net, the model utilized a loss function defined as binary cross‐entropy with logits. Optimization was carried out using the Adam optimizer. The training regimen involved iterating over the entire dataset for a total of 20 epochs, with the objective of minimizing the loss to a substantially low value.

### Tumor Segmentation Procedures

#### Manual Labeled Data

For each rabbit tumor scan, the collected RF data were converted into B‐mode images for later processing. Those scans were evaluated qualitatively based on the perceived image quality, and the scans with distinct tumor boundaries and visible internal tissue structures, ensuring that the tumor interiors were not empty and suitable for BSC calculations, were chosen. In each scan, the array probe was physically translated elevationally across the tumor to acquire a tumor volume of image data. For each tumor scan, we selected the scan exhibiting the highest bead signal to serve as the reference frame and to act as the center of the tumor volume to be processed. In total, 10 frames were selected for each tumor. Sequential frames represented imaging of different planes in the tumor as the probe moved elevationally. Ten frames (5 on the left side and the other 5 on the right side of the bead frame) were selected at even spacing in the frame number in the elevational direction spanning from the edge of the tumor on one side of the bead frame (5 slices), the bead frame and then an additional 4 frames were selected from the bead frame to the other edge of the tumor. These 10 frames collectively spanned the entire tumor, ranging from one edge to the other edge of the tumor. The tumor regions in these frames were hand labeled as the ground truth. The segmentation process was conducted by a single expert reader trained in ultrasound imaging and familiar with tumor morphology, to ensure accuracy and consistency throughout the dataset. For each selected tumor frame, the tumor boundaries were carefully outlined based on visual inspection of the ultrasound images, ensuring consistent delineation across samples. The region within each outlined boundary was assigned a mask value of 1 (indicating the presence of tumor tissue), while the regions outside the boundary were assigned a value of 0 (non‐tumor regions). Through this method, we collected a total of 2198 manually labeled scan frames.

#### U‐Net‐Based Segmentation

For our deep learning‐based segmentation, the U‐Net architecture was utilized. For the total 2198 images collected from 21 rabbits, the dataset was randomly split into 16 rabbits for training, 2 rabbits for validation, and 3 rabbits for testing. To enhance the diversity of the training data, we employed traditional data augmentation techniques including rotation, flipping, and elastic deformation. All the B‐mode images and corresponding labeled tumor region images were down‐sampled to 256 × 256 using the cv2.resize function in Python to accelerate the U‐Net processing. The validation set served as a tool to monitor training progress and avoid overfitting. After the training, the U‐Net model was assessed through the testing images, which were divided into 2 groups: with and without fat overlay, to evaluate whether the additional losses from the fat overlay layer introduced any differences. Accuracy, precision, recall, and Dice Score were calculated using the corresponding hand‐labeled tumor regions as the ground truth. The whole process was repeated across 4 different splits, that is, 4‐fold validation, to ensure robustness and reliability of the model's performance, where the detailed information is shown in Table 1.

**TABLE 1:** Number of Training, Validation, and Test Images Across Data Splits

Data Split	Training Images	Validation Images	Test Images (No Fat Overlay)	Test Images (With Fat Overlay)
1	1679	268	148	103
2	1677	210	170	141
3	1733	258	114	93
4	1628	209	196	165

### Bead Localization

In order to use the bead as an in situ reference, imaging frames containing the bead and the actual bead location in the imaging frames must be detected for processing the bead signal. Furthermore, the bead location is different in each tumor image frame, and the accidental inclusion of the bead in a data block used to calculate the BSC for the tumor could result in errors in the BSC estimated for the tumor and subsequent parameters derived from the BSC. Therefore, the bead localization is needed for every frame. Manually selecting the bead location from many image frames is a laborious and challenging task, requiring individuals to scan through all the frames, identify a possible frame, and label the bead location. Automated bead identification and separation can significantly streamline the entire BSC data processing pipeline.

**Figure 2:**
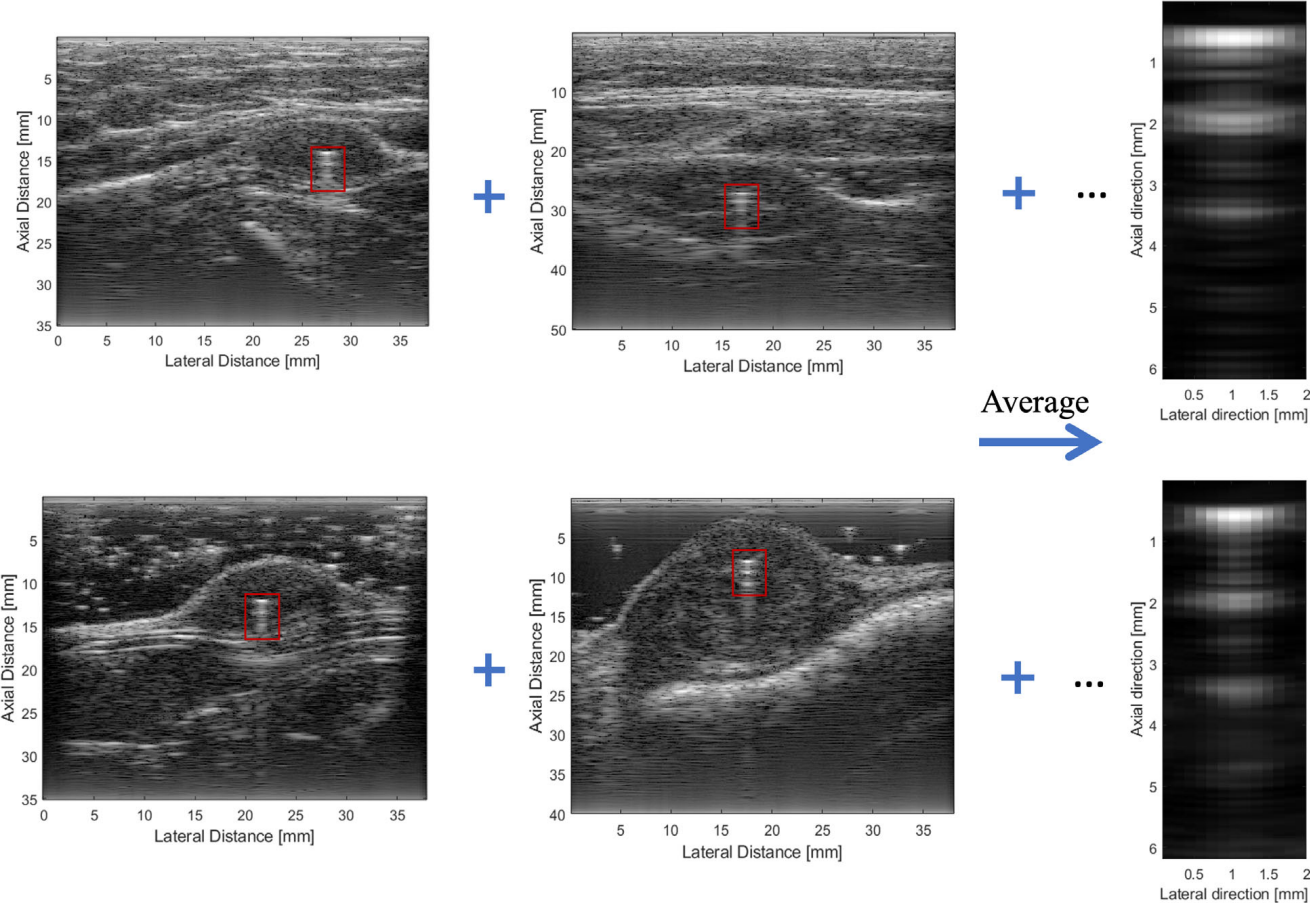
B‐mode imaging of bead BSFs (right) extracted from 6 sets of labeled bead data: with (top) and without (bottom) pork belly overlay.

In previous research, the automatic detection of the bead in the images was accomplished by correlating the bead locations in frames containing beads with a reference bead signal.^18^ First, a bead scattering function (BSF), which was extracted from 6 sets of labeled bead data (shown in Figure 2), was selected as a reference. The BSF for the bead includes reverberation from multiple echoes and is consistent between cases where more attenuation of the signal occurs, that is, when a fatty layer is on top of the sample. Next, the RF data for different imaging frames were converted to B‐mode images using a decibel (dB) scale with a dynamic range of 60 dB. The B‐mode images were processed by normalized cross‐correlating (NCC) each frame with the BSF. The maximum coefficient value and its location were recorded for each frame, and this process was repeated for all frames in a tumor data set. Finally, the frame with the maximum cross‐correlation coefficient was labeled as the desired frame for calculating the bead reference signal.

This previous bead localization procedure did not perfectly identify the bead locations.^18^ Specifically, in some cases, there existed small structures inside or outside the breast tissue that looked like the bead signals, that is, with a strong main echo and subsequent several smaller echoes. These structures could result in mislabeling and reduce the accuracy of bead localization. Because of the tumor segmentation, the cross‐correlation can be constrained to be inside the tumor region, which is more uniform and contains fewer structures like the bead. This added constraint is hypothesized to improve the bead localization accuracy.

### Quality Metrics for the Segmentation

#### Dice Score

In the realm of image segmentation, the Dice score, also known as the Dice similarity coefficient (DSC), is a metric used for evaluating the accuracy of segmentation models. Defined mathematically as twice the area of overlap between the predicted segmentation and the ground truth, divided by the total number of pixels in both the predicted and ground truth segmentation, it provides a measure of the model's performance,

(1) $$\text{Dice Score}=\frac{2\times \left|P\cap G\right|}{\left|P\right|+\left|G\right|}.$$

The Dice score ranges from 0 to 1, where 1 signifies perfect agreement between the model's prediction and the ground truth. The Dice score is mathematically equivalent to the F1 score, which is a harmonic mean of precision and recall. This metric is especially valuable in scenarios where the classes are imbalanced, as it accounts for both false positives and false negatives. It offers a balanced view of the model's ability to accurately segment images, making it a standard choice for assessing medical image segmentation tasks.

#### Accuracy, Precision, and Recall

In the domain of machine learning and statistical modeling, the assessment of a classification model's performance is necessary for ascertaining its efficacy in predictive analysis. Four principal metrics, namely accuracy, precision, recall, and the F1 score, are extensively employed for this evaluation, each offering distinct insights into the model's performance characteristics. For this binary tumor segmentation task, the F1 score is the same as the Dice score.

Accuracy measures the overall correctness of model predictions. Precision calculates the accuracy of positive predictions, vital when false positives are costly. Recall evaluates the ability to identify all actual positives, crucial in areas like medical diagnostics to avoid missing critical detections.

(2) $$\begin{array}{c} \text{Accuracy}=\frac{\mathrm{TP}+\mathrm{TN}}{\mathrm{TP}+\mathrm{TN}+\mathrm{FP}+\mathrm{FN}}\\ \text{Precision}=\frac{\mathrm{TP}}{\mathrm{TP}+\mathrm{FP}}\\ \begin{array}{c} \text{Recall}=\frac{\mathrm{TP}}{\mathrm{TP}+\mathrm{FN}}\\ F1=2\times \frac{\text{Precision}\times \text{Recall}}{\text{Precision}+\text{Recall}}, \end{array} \end{array}$$

where *TN* means true negative, *TP* means true positive, *FN* means false negative and *FP* means false positive.

### Extraction of QUS Parameters

For each frame, the segmented tumor region (both hand labeled and U‐Net segmented) was separated into small data blocks (3 mm × 3 mm, around 10 pulse lengths per side) with 75% overlap and the average BSC of each tumor region was calculated using the bead reference method, where the bead was injected into the tumor. The in situ bead reference assumed that the transmission and attenuation loss of tissue at the location of bead was approximately the same as the transmission and attenuation loss of adjacent tissues at the same depth as the bead. In addition, the backscattered signal from the bead was much larger than the signal from the surrounding tissue. Under these 2 assumptions, the BSC for a sample can be estimated from the backscattered power spectrum calculated from the bead:

(3) $$\sigma \left(f\right)=\frac{W\left(f,\vec{x}\right)}{W_{\text{bead}}\left(f,\vec{x}\right)}\sigma_{\text{bead}}\left(f\right),$$

where $W_{\text{bead}}\left(f,\vec{x}\right)$ and $W\left(f,\vec{x}\right)$ are the bead and tumor power spectra and $\sigma_{\text{bead}}\left(f\right)$ and $\sigma \left(f\right)$ are the bead and tumor BSCs, respectively.^27^ Attenuation and transmission losses in the tissue layers, which are assumed to be the same for the bead, are eliminated by division. Because the BSC is system and operator independent, the BSC of a bead of particular material and size only needs to be calculated once for all experiments. The BSC of a 2‐mm Ti bead was estimated by measuring the ultrasound backscattered signal from a bead embedded in a tissue‐mimicking phantom.^18^

Two scatterer‐related parameters, ESD and EAC, were estimated from the BSC using a spherical Gaussian scattering model^11^, ^28^

(4) $$\sigma =\frac{\bar{n}k^{4}V_{s}^{2}\gamma_{0}^{2}}{16\pi^{2}}e^{-2k^{2}d^{2}},$$

where σ is the BSC, $\bar{n}$ is the average number density of particles, *k* is the wave number, *d* is the characteristic dimension, $V_{s}={\left(2\pi d^{2}\right)}^{3}$ is the average particle volume, and $\gamma_{0}=\left(Z-Z_{0}\right)/Z_{0}$ is the fractional change in impedance between the scatterers and the surrounding medium. For $\gamma_{0}$, *Z*, and *Z*_0_ are the scatterer impedance and the surrounding medium impedance, respectively.

The relationship between ESD and characteristic dimension *d* is: ESD = 3.11*d*, and the product of $\bar{n}$ and $\gamma_{0}^{2}$ is called the EAC. The estimates ESD and EAC provide a physical interpretation of the tumor microstructure.

### Automatic BSC Estimation

Combing the U‐Net tumor segmentation, bead localization, and the BSC estimates, we could automate the BSC estimation following the simple protocol. 1) RF files were first converted into B‐mode images, which were then processed by the U‐Net to identify tumor regions. 2) By integrating the BSF NCC algorithm with the segmented tumor regions from the U‐Net, we were able to automatically locate the reference bead. This bead served as a reference point for BSC calculation. 3) BSCs were then calculated for each data block in the segmented tumor, and estimates of ESD and EAC were also estimated for each data block from the corresponding BSCs. Only data blocks without an identified bead were used in the calculations of the BSC in the tumors.

## Results

### Rabbit Breast Tumor Segmentation

**Figure 3:**
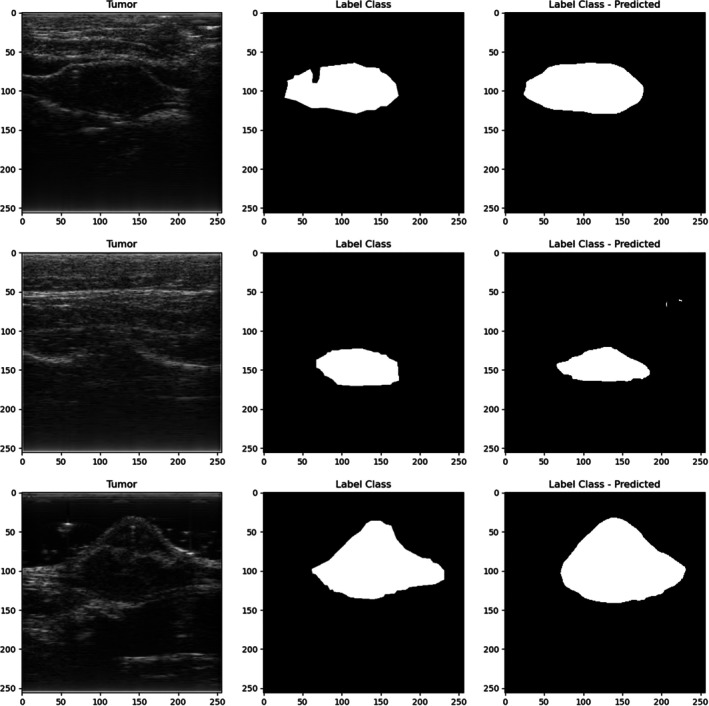
B‐mode images (left), hand‐labeled tumor regions (middle), and predicted tumor regions (right).

Figure 3 showcases 3 test images as an illustration of U‐Net's tumor detection capabilities. Generally, the U‐Net successfully identified tumor regions, though its accuracy was not flawless. As demonstrated in the bottom image, the predicted tumor area could sometimes be larger than the manually selected tumor. The middle image highlights instances where non‐tumor locations were also erroneously labeled as part of the tumor. A notable example is the top image, where a discrepancy was observed between the U‐Net prediction and the manual labeling. In the hand‐labeled image, a gap was deliberately left near the tumor surface to accommodate a bead, whereas the U‐Net prediction overlooked the bead's presence, resulting in a continuous tumor region without this gap. Across the 4 sets of test images using different splitting methods, the evaluation metrics demonstrated high consistency between the hand‐labeled ground truth and the U‐Net predicted images, as well as consistency among the 4 different split test sets. This highlights the U‐Net's robust capability in predicting tumor images, demonstrating its independence from variations in data splitting. Moreover, the differences between the “no fat overlay” and “fat overlay” conditions were minimal. Across the 4 different split sets, the differences in the Dice Scores between segmentation with or without a fat overlay layer was below 0.05, indicating that the U‐Net model performed consistently regardless of the presence of fat overlay. To assess whether the presence of additional overlying tissue significantly affected segmentation performance, we performed Kruskal‐Wallis tests comparing the “fat overlay” and “no fat overlay” conditions across 4 evaluation metrics. The results showed no statistically significant differences (*P* > .05), although a trend toward significance was noted in recall (*P* = .0833), suggesting a possible influence of attenuation on the model's sensitivity to tumor regions. The evaluation metrics for all 4 test sets are summarized in Table 2. Due to the consistency of the performance metrics observed across the 4 different splits, we utilized the data from splitting method one, consisting of 251 images (148 with no fat overlay layer and 103 with fat overlay layers) for subsequent analyses, which includes computation time comparison, bead localization, and QUS parameter estimation. This choice was made to streamline the discussion without sacrificing generality.

**TABLE 2:** Performance Evaluation Metrics of the U‐Net Model Across 4 Test Scenarios

	Accuracy			Recall			Precision			Precision		
	No^a^	With^b^	All^c^	No	With	All	No	With	All	No	With	All
1	0.9786	0.8894	0.8718	0.8702	0.9808	0.8929	0.8948	0.8878	0.9795	0.8908	0.8812	0.8775
2	0.9722	0.8577	0.9227	0.8842	0.9672	0.9189	0.7870	0.8367	0.9699	0.8854	0.8612	0.8627
3	0.9638	0.9086	0.7976	0.8303	0.9834	0.9489	0.8398	0.8837	0.9726	0.9267	0.8166	0.8543
4	0.9590	0.8313	0.8188	0.8132	0.9749	0.8995	0.8271	0.8456	0.9663	0.8606	0.8226	0.8280
Overall	0.9684	0.8718	0.8527	0.8495	0.9766	0.9151	0.8372	0.8635	0.9721	0.8909	0.8454	0.8556

^a^ Test images without a fat layer.

^b^ Test images with a fat layer.

^c^ All test images, with and without a fat layer.

**Figure 4:**
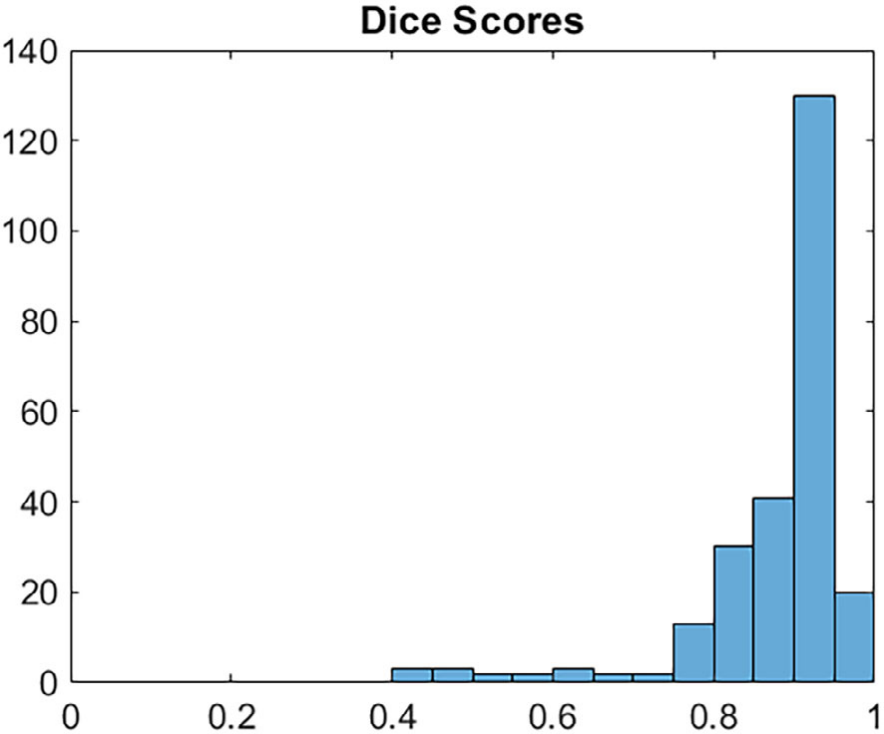
Histogram of Dice Scores across 221 test images.

**Figure 5:**
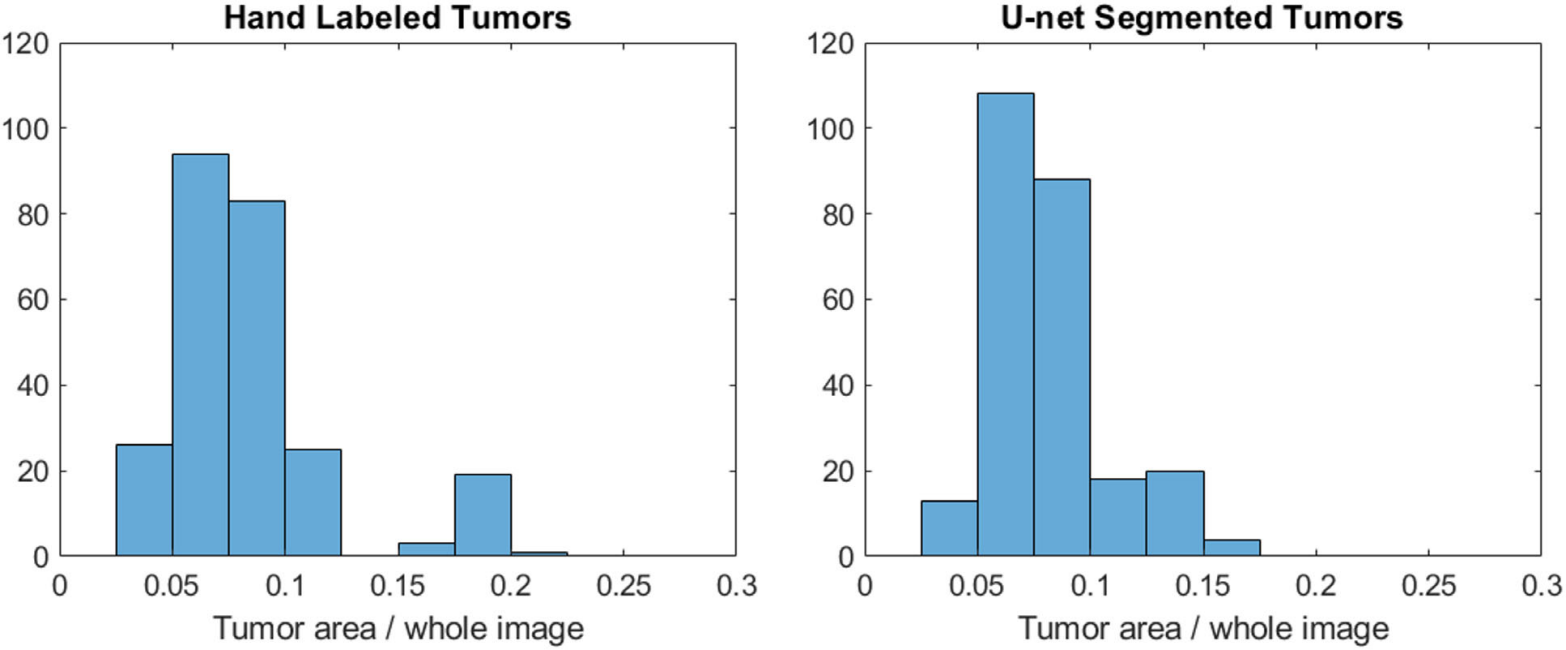
Comparative histograms of tumor‐to‐image area ratios: Hand‐labeled (left) versus U‐Net segmented (right) tumor regions.

The Figure 4 presents a histogram of the Dice Scores for all 251 test cases. It indicates that 150 sets had a Dice score greater than 0.9, and 221 sets had a Dice score exceeding 0.8, demonstrating effective tumor segmentation performance. Considering that a perfect match between the U‐Net segmented tumor regions and manually labeled regions is unattainable due to the inherent approximation in hand‐labeling and the possibility of human error, these scores emphasize the effectiveness and reliability of U‐Net for the rabbit tumor image segmentation. Figure 5 illustrates that the U‐Net struggled to accurately predict small and large tumors, specifically those with a tumor‐to‐image area ratio smaller than 0.05 or bigger than 0.175.

### Bead Localization

**Figure 6:**
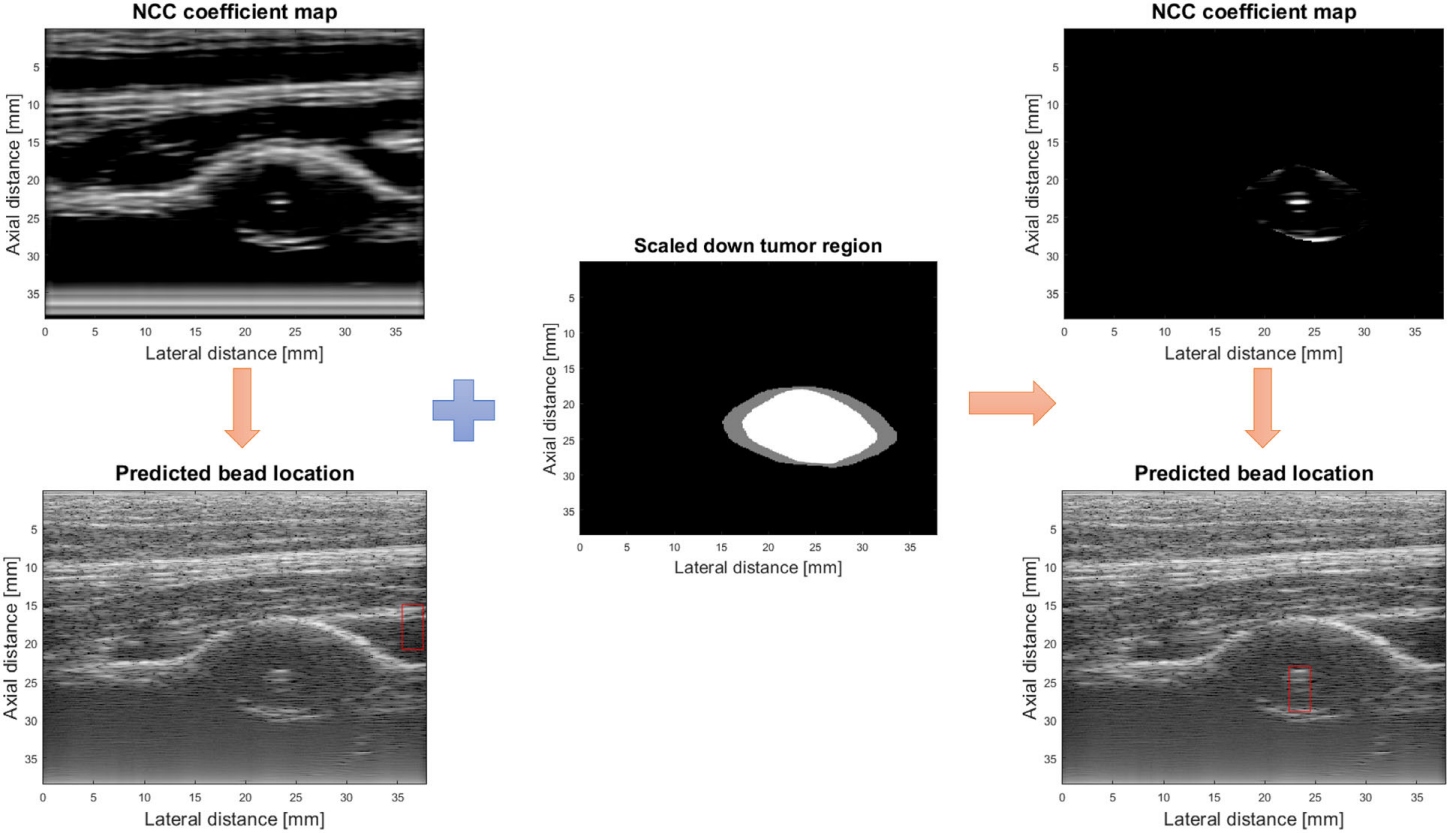
NCC map of the entire image with the incorrectly labeled bead location (on the left); predicted tumor region alongside the tumor's internal area (in the middle); and the NCC map focusing on the tumor's internal area with the correctly identified bead location (on the right).

Figure 6 illustrates the role of the tumor region in accurately localizing the bead. In the left image, the bead was incorrectly identified using the NCC method. This mislabeling occurred because a region outside the tumor, resembling the bead, led to a high NCC coefficient. However, when the NCC was applied only within the tumor's interior, the bead was correctly located. This improved accuracy can be attributed partly to the more uniform nature of the tumor's interior, which makes the bead easier to distinguish and accurately identify compared to other areas.

**Figure 7:**
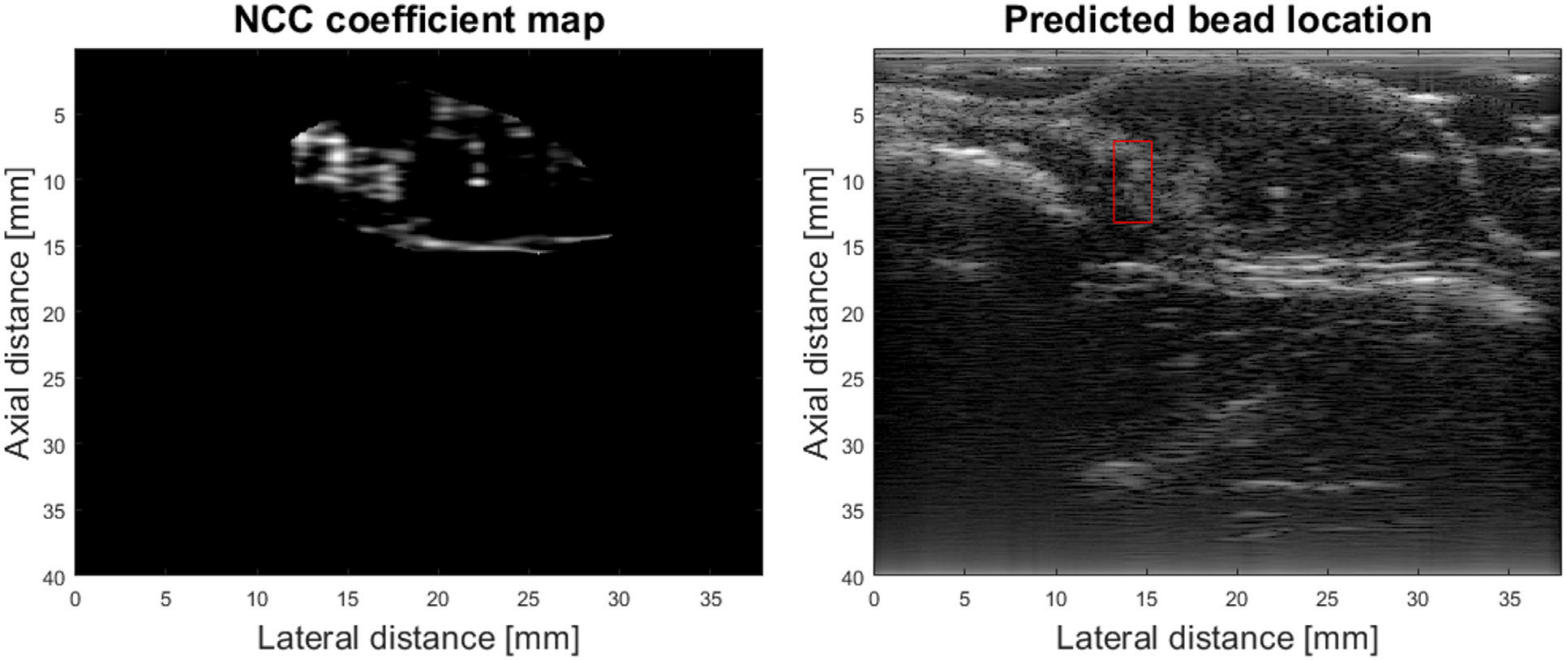
NCC map inside the tumor region (left) and incorrectly labeled bead location (right).

We also tested the accuracy of the bead localization method on the 251 test sets. With the BSF NCC method only, the beads in 34 frames were mislabeled. When including the search only within the segmented tumor region, the number of incorrectly labeled bead frames decreased to 14. The new bead detection method successfully eliminated more than half of the mislabeled images, improving the accuracy. The accuracy of bead detection increased from 86.45 to 94.42%, suggesting that the U‐Net tumor region segmentation method could not only help the BSC estimate but also help localize the bead, facilitating an automatic and potentially real‐time BSC estimate and mapping. Among the 14 frames that were incorrectly labeled, the bead signal was too faint for the BSF method to accurately identify the bead's location, as demonstrated in Figure 7.

### QUS Parameters: Automatic Versus Human‐Segmented Tumors

**Figure 8:**
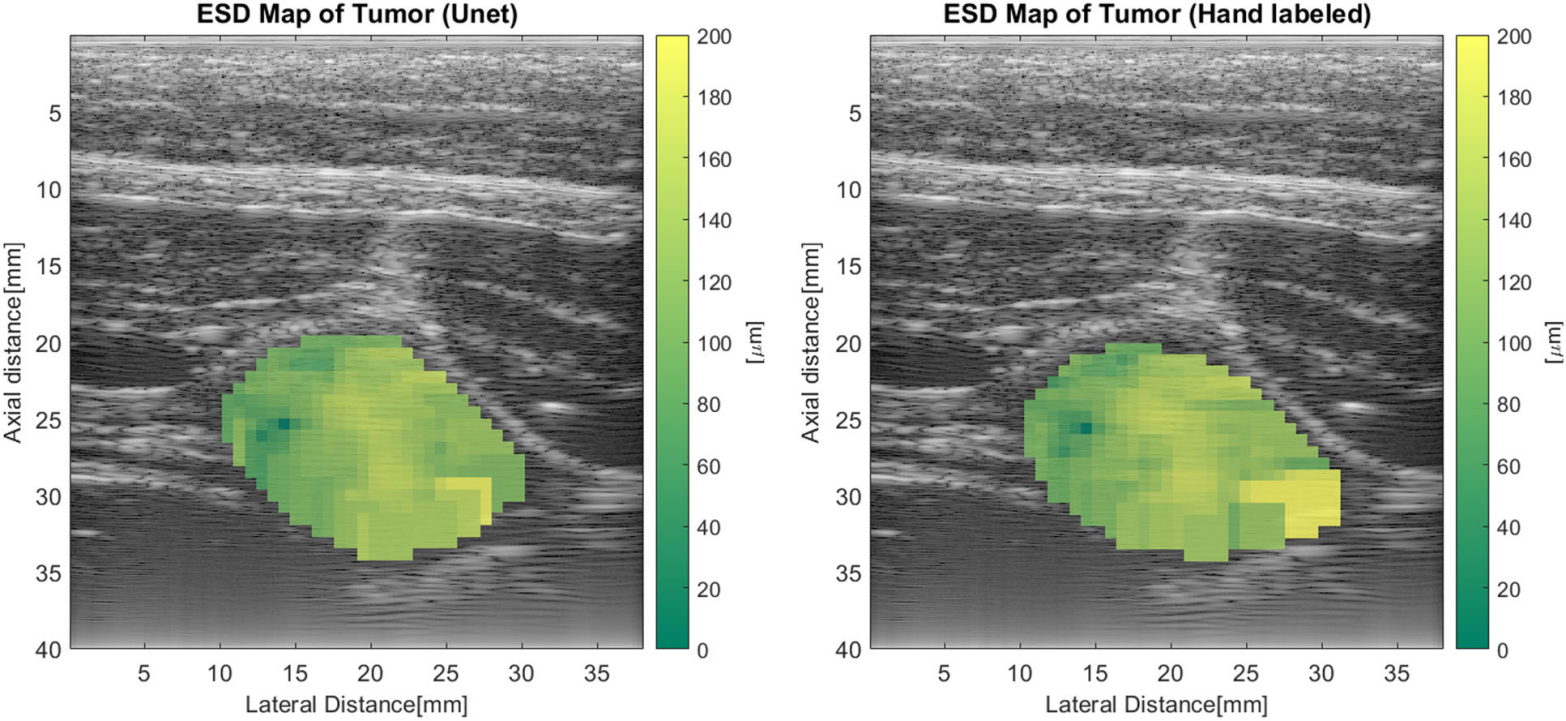
ESD map of the U‐Net segmented tumor region (left) versus the hand‐labeled tumor region (right).

Figure 8 presents a comparison between the ESD map of a tumor segmented by the U‐Net and that of a tumor region labeled manually. The similarity in the ESD maps between these two images suggests that the U‐Net can accurately segment tumor images and generate reliable QUS maps. We then calculated the relative error of the averaged ESD and EAC, between the U‐Net and human‐segmented tumors. The average ESD and EAC values were computed by averaging the values from all separated 3 mm × 3 mm boxes from tumors, excluding those with ESD values equal to 1, as such boxes did not fit the Gaussian model of BSC. The relative error is defined as:

(5) $$\text{Relative error}=\frac{\left|{\text{Value}}_{U-\mathrm{net}}-{\text{Value}}_{\text{human}}\right|}{{\text{Value}}_{\text{human}}}.$$

**Figure 9:**
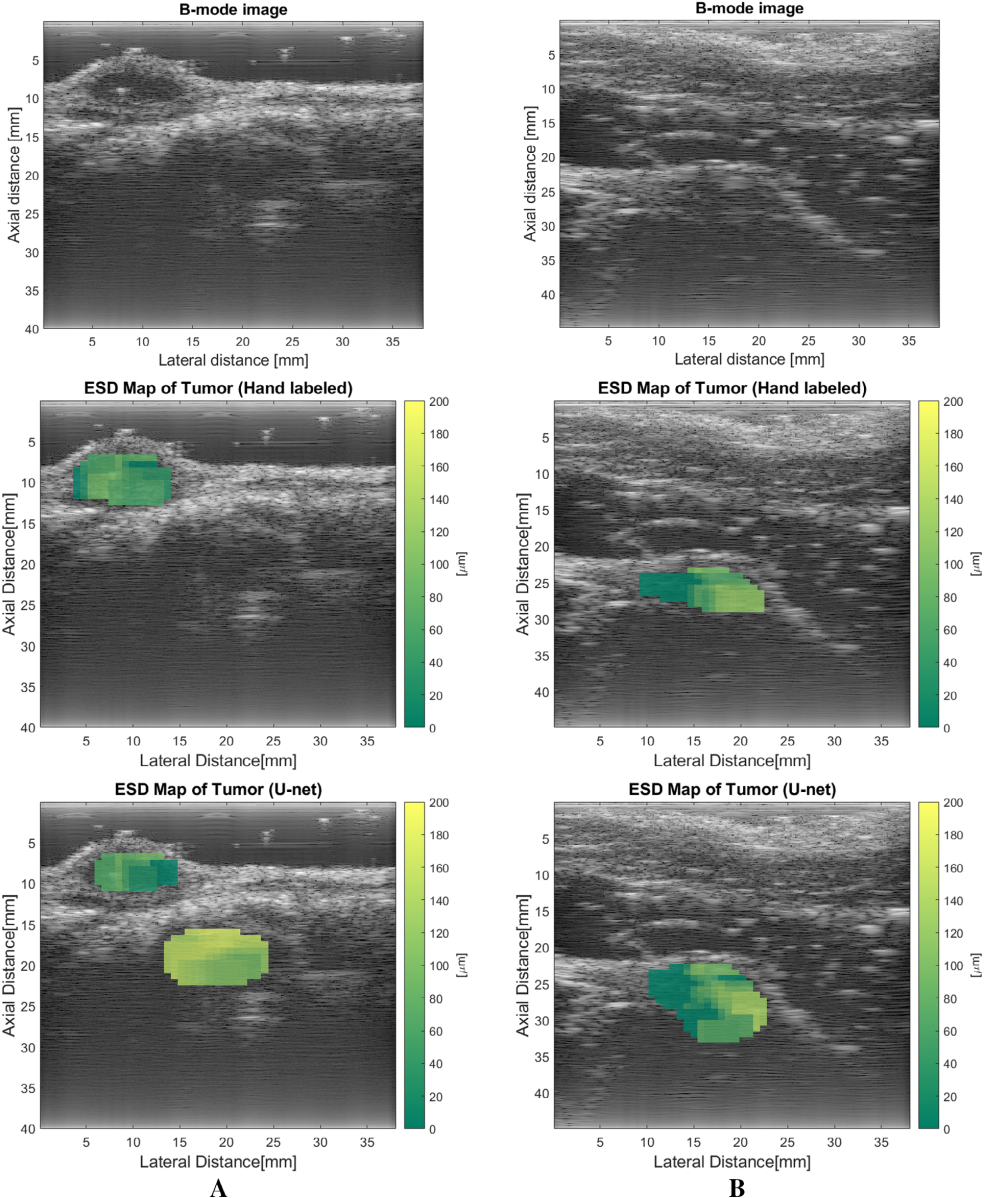
ESD maps of tumor segmented by hand (middle) and by U‐Net (bottom).

For all the 251 test sets, the average ESD relative error was 17.87%, and the average EAC relative error was 9.95%. The primary source of the error came from one tumor scan where the U‐Net incorrectly identified non‐tumorous tissue as tumor. An instance of this error is illustrated in Figure 9A. After we omitted this set, the average ESD relative error reduced to 9.30%, and the average EAC relative error was lowered to 6.67%. Furthermore, the U‐Net often failed to delineate tumor edges as precisely as manual labeling, shown in Figure 9B, where the layer surrounding the tumor, possessing distinct parameters compared with the tumor, contributed to inaccuracies in BSC estimates. This also indicates that the U‐Net is not good at detecting small tumors, as we have discussed before.

## Discussion

The results of the U‐Net for automatic rabbit mammary tumor segmentation and subsequent BSC estimation demonstrated the algorithm's effectiveness and efficiency, highlighting its potential to streamline traditional manual methods and enable real‐time applications. Once trained, this approach can significantly reduce the time and effort needed for tumor segmentation. The investigation underscores the inherent challenges in precise tumor segmentation and suggests that automated techniques could augment human accuracy and expertise.

Although a U‐Net is commonly used for tumor segmentation, in this study, the segmented tumor regions obtained using U‐Net were utilized for BSC estimation, representing a novel approach. The ESD and EAC results were consistent with manually labeled data, even in low SNR images, which sometimes led to segmentation inaccuracies. In cases where tumor boundaries were not clear, even experienced experts may need extra time, leading to potential errors. Consequently, the U‐Net may occasionally fail to accurately segment tumors under these conditions, underscoring the need for continuous refinement of deep learning models and continued human oversight.

Using the in situ Ti bead as a reference for BSC calculations compensates for attenuation and transmission losses but introduces the challenge of bead localization, which requires additional time. Previously, we used the NCC across entire images for bead detection, which occasionally misidentified non‐tumor tissues resembling the bead.^18^ Limiting NCC to within the tumor region improved bead localization accuracy from 86.45 to 94.42%, reducing manual verification and enhancing the method's reliability for real‐time use. Limitations arose when bead signals were weak, leading to mislocalization. A potential solution is to utilize multiple frames to aggregate more data on the tumor and bead location, enhancing accuracy. Consequently, the U‐Net's input could consist of multiple B‐mode images instead of a single frame.

The U‐Net significantly accelerated tumor segmentation, processing 251 test images in only 39.91 seconds, reducing manual labor and suggesting the feasibility of real‐time BSC estimation during imaging. While initial investment is needed for network training, the trained model can be used across various rabbit tumor scans, showcasing its utility in diagnostic workflows.

Leveraging these advancements, automatic BSC estimation from tumors segmented by the trained U‐Net was achieved. Constrained NCC was used to localize the reference bead within each scan, identifying the bead with the strongest signal. Segmented tumor regions were divided into boxes of specific sizes for QUS imaging. The rapid processing by U‐Net enables potential real‐time application, as manual segmentation is unnecessary during the exam.

Testing the U‐Net under varied imaging conditions, such as low SNR scenarios, is essential. While current scans yielded high SNR and contrast, imaging through obstructions like bandages may degrade image quality, affecting segmentation accuracy. Incorporating other deep learning strategies could enhance U‐Net robustness, ensuring precise segmentation even under challenging conditions.

While the training dataset consists of 16 rabbits, which is relatively small by deep learning standards, each rabbit provides multiple imaging frames, increasing the effective dataset size. Additionally, data augmentation strategies were employed to improve generalization. The current model is intended as a foundational segmentation framework, with the goal of incrementally enhancing performance through continued data acquisition. As we transition toward clinical use, we envision applying transfer learning techniques to adapt the model to human data. This strategy allows the model to evolve over time by incorporating new patient scans, ultimately building a robust, continuously improving segmentation system for diverse clinical scenarios.

VX2 tumors exhibit a broad range of tissue structures that represent a broad distribution of potential scatterer sizes, from single cancer cells on the order 20–30 μm, to multicellular arrangements or clusters of cells (50–100 μm or larger), to interfaces between histologically distinct regions (eg, viable versus necrotic tissue having variable sizes >100 μm). The average ESD estimated from the all of the rabbit tumors was 72.6 ± 21.2 μm based on using a spherical Gaussian model, which is often chosen when the model for scattering is not known. The ESD estimate is a function of the different scattering sources within the scattering volume. Because identified structures are diverse in size, the ESD estimate represents a biased estimate of the underlying structure.

Future work will focus on optimizing segmentation algorithms to minimize errors and integrating these advanced techniques into clinical settings. This will enhance diagnostic and treatment planning with more accurate and timely data interpretation. Testing U‐Net on additional human data will also be explored and compared to the rabbit tumor model.

## Conclusions

This study demonstrated the effectiveness of U‐Net for automatic segmentation of rabbit mammary tumors and to improve calibration bead identification to facilitate BSC estimation. By training on rabbit mammary tumor images collected from 16 rabbits and testing on images from 3 rabbits, the U‐Net demonstrated robust performance, significantly reducing the manual effort required for tumor segmentation. Furthermore, the test results across 4 different splitting methods highlighted the consistency and reliability of the U‐Net, irrespective of the splitting strategy. The use of tumor segmentation also improved the ability to automatically detect the correct location of a calibration bead, reducing the number of incorrect classifications by almost half. The promising results indicate potential for real‐time BSC calculation when using an in situ calibration target, which could enhance QUS applications by enabling immediate feedback and improving clinical workflows in tumor characterization.

## Data Availability

The data that support the findings of this study are available from the corresponding author upon reasonable request.
